# Supplementary material for: The discovery of novel noncoding RNAs in 50 bacterial genomes
Source: Nucleic Acids Res. 2024 Apr 22;52(9):5152–65. doi: 10.1093/nar/gkae248 (PMC11109978; doi:10.1093/nar/gkae248)
Supplement: gkae248_Supplemental_Files [file gkae248_supplemental_files.zip › Narunsky 50 Genomes RNA Motifs Files.zip!/Publication/55-Hydrogenobaculum_sp_Y04AAS1/Figures/LRC-55-5-NC_011126_1_IGR_51_277914_278069.sto.pdf]

LRC-55-5-NC_011126_1_IGR_51_277914_278069.sto.r2r

5′-○○○○○○○○○○CCCCCUUUUUGGGGGUUUAUAAY●AUAUUAA●AUAU●UY●●●●●●●●●R●●●AAAAR●●R●AAAAGCC○●YUAUGGG○RGGCUUUUACA○AGGAGGAUAAAGCUAUGAAAAGACAAAAGGCUCUC●●●●GAGAGCCYUAACAAC
